# Girl Who Cried Wolf: A Case of Prinzmetal Angina With Related ST-Elevation Myocardial Infarction

**DOI:** 10.7759/cureus.12661

**Published:** 2021-01-12

**Authors:** Aahana Gaur, Saikrishna Patibandla, Sandeep Sohal, Constantine Monzidelis, Samir Garyali

**Affiliations:** 1 Internal Medicine, The Brooklyn Hospital Center, Brooklyn, USA; 2 Cardiology, West Virginia University, Morgantown, USA; 3 Cardiology, The Brooklyn Hospital Center, Mount Sinai Hospital, Brooklyn, USA; 4 Cardiology, The Brooklyn Hospital Center, Brooklyn, USA

**Keywords:** prinzmetal, non-obstructive coronary artery disease, st-segment elevation, prinzmetal angina

## Abstract

Prinzmetal variant angina is characterized by episodic chest pain associated with transient ST changes seen on an electrocardiogram (EKG). A 51-year-old female with a pertinent history of non-obstructive coronary artery disease (CAD), non-ST elevation myocardial infarction (NSTEMI) twice, ST-elevation myocardial infarction (STEMI), Prinzmetal angina, ventricular tachycardia s/p implantable cardioverter-defibrillator placement, and gastroesophageal reflux disease presented with 2.5 hours of left-sided chest pain with radiation to the left arm. Her initial EKG was not revealing. However, a subsequent EKG showed ST elevations in the inferior leads. A coronary angiogram was performed and revealed distal right coronary artery spasm that was relieved with intracoronary nitroglycerin. The nature of her chest pain in conjunction with her EKG and angiogram findings helped diagnose her with Prinzmetal angina that was significant enough to result in a STEMI. Thus, Prinzmetal angina and STEMI can be interconnected rather than being separate, mutually exclusive pathologies.

## Introduction

Prinzmetal (vasospastic) variant angina was first described by Myron Prinzmetal in 1959 as a form of chest pain that occurred at rest or ordinary activity but not strenuous exercise [1]. It is seen more commonly in Japanese patients than other populations, and more often in men within the age of 40 to 70 years of age; the prevalence drops after age 70 [2]. The coronary vasomotion disorders international study (COVADIS) group attempted to generate standardized criteria for diagnosing vasospastic angina based on three key elements: 1) spontaneous angina that responds to nitrates, 2) transient electrocardiogram (EKG) changes during a spontaneous anginal episode, 3) documented coronary artery spasm with angina and ischemic EKG changes either spontaneously or in response to provocation testing during angiography [2]. Prinzmetal angina occurs typically between midnight and the early morning with transient ST-T wave changes on EKG corresponding to an epicardial coronary artery experiencing total or subtotal spasm during an acute episode of chest pain [3]. However, it can be difficult to diagnose as patients may have asymptomatic or atypical episodes that lack certain typical features suggestive of Prinzmetal angina. One estimate states that only 20-30% of patients report chest pain when they have coronary vasospasm and ischemic ST-segment elevation on EKG [4]. We describe the case of a patient with recurrent, refractory Prinzmetal angina despite being on maximal antianginal therapy who subsequently developed myocardial infarction (MI).

## Case presentation

The patient is a 51-year-old woman with a pertinent history of stable angina (diagnosed nine years prior), Prinzmetal angina (diagnosed two years ago with vasospasm in the right coronary artery relieved with intracoronary nitroglycerin), non-obstructive coronary artery disease (CAD) characterized by a non-ST elevation MI (NSTEMI) treated medically one and two years ago, ventricular tachycardia s/p implantable cardioverter-defibrillator placement nine years ago with replacement two years ago, heroin abuse on methadone and gastroesophageal reflux disease. Pertinent home medications were aspirin 81 mg daily, rosuvastatin 40 mg daily, isosorbide mononitrate 120 mg twice daily, ranolazine 1000 mg twice daily, metoprolol tartrate 100 mg twice daily, and verapamil 360 mg nightly. She initially presented to the emergency department (ED) with complaints of left-sided chest pain that started at rest, radiated to the left arm, and lasting approximately 150 minutes. Her vital signs were a blood pressure of 159/82 mmHg, heart rate of 71 beats per minute, respiratory rate of 22 breaths/minute, and oxyhemoglobin saturation of 97% on room air. An initial evaluation in the ED revealed an abnormal EKG with Q waves in the inferior leads, a prolonged QTc of 530 ms, and no ST-T wave changes. The initial troponin level was 0.01 ng/mL. Her brain natriuretic peptide level on admission was 69 pg/mL (normal: <100 pg/mL). Her lactic acid was elevated at 2.7 (normal: <2.0), and her creatine kinase-myocardial band (CK-MB) level was normal. A urine drug screen was positive for methadone and negative for other illicit drugs, including cocaine.

**Figure 1:**
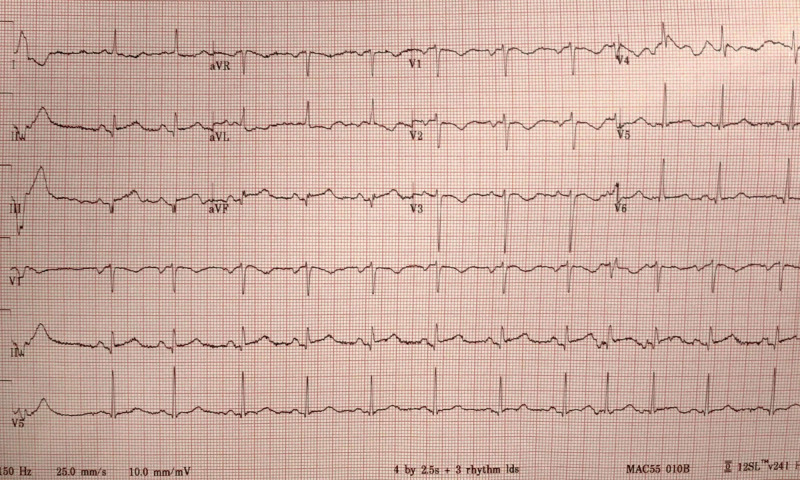
EKG showing ST elevations in the inferior leads and QT prolongation EKG - electrocardiogram

**Figure 2:**
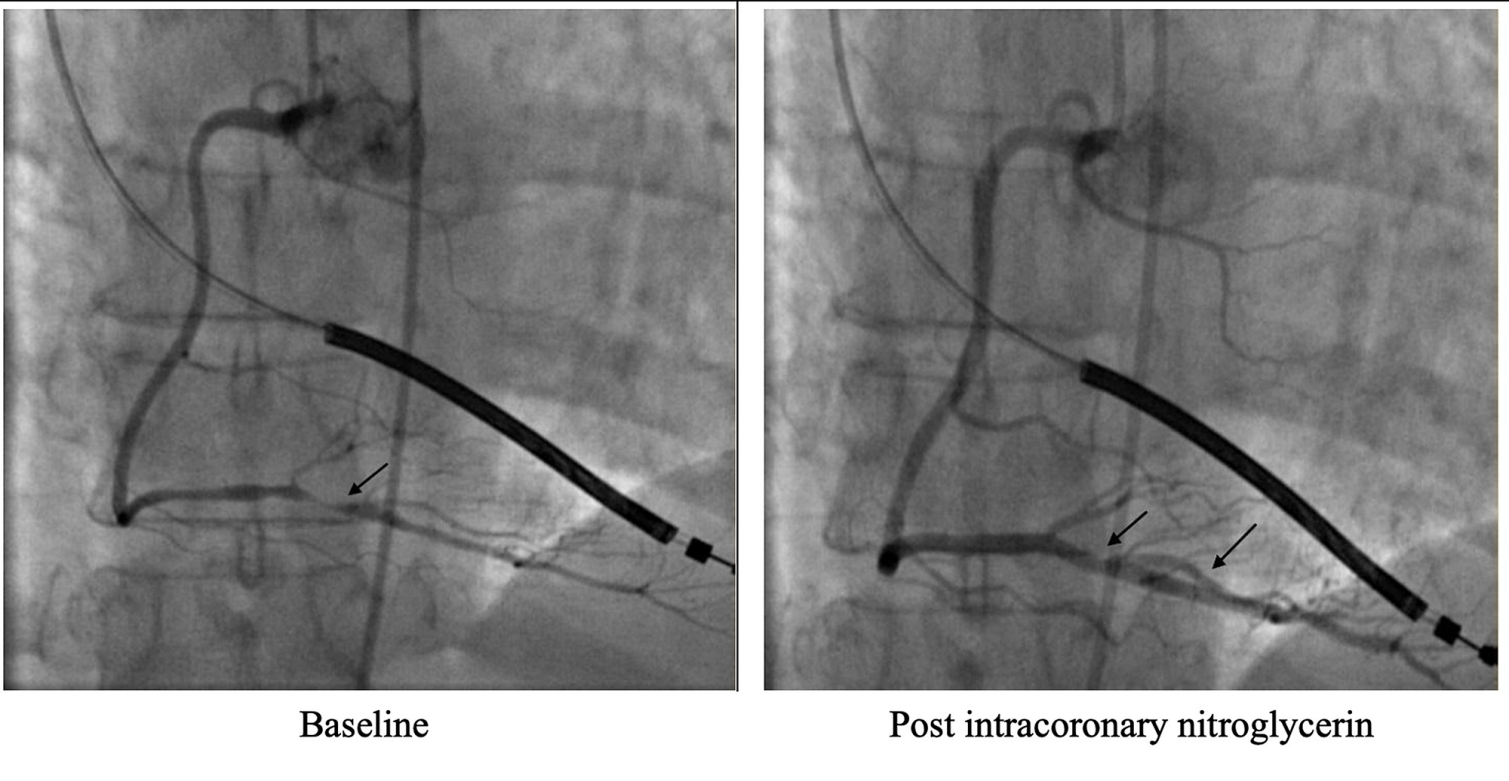
Coronary angiography before and after intracoronary nitroglycerin Coronary angiogram showing a 95% lesion (black arrow) in RPDA (LAO view [left]). Resolution of RPDA disease segment after administration of IC nitroglycerin consistent with coronary artery spasm (black arrow; LAO view [right]). RPDA - right posterior descending artery; LAO - left anterior oblique; IC - intracoronary

With the presence of recurrent chest pain, she was treated with sublingual nitroglycerin tablets without any relief. A repeated EKG done three hours later showed an ST-segment elevation in leads II, III, and augmented vector foot EKG lead (aVF) with corrected QT interval​​​​​​​ (QTc) > 500 ms (Figure 1). At this time, vital signs were a blood pressure of 184/162 mmHg, heart rate of 76 beats per minute, respiratory rate of 22 breaths per minute, and oxyhemoglobin saturation of 98% on room air. These remarkable EKG changes prompted the change in the management plan, and an emergent cardiac catheterization was performed. The catheterization revealed a non-obstructive left coronary artery system and a right coronary system demonstrating a 95% stenosis of the distal right coronary artery (RCA). Given the patient's medical history and clinical presentation, suspicion for coronary vasospasm was high on a differential. Subsequently, intracoronary nitroglycerin was given with complete resolution of distal RCA occlusion, consistent with coronary vasospasm (Figure 2). The left main coronary artery was an ectatic vessel with luminal irregularities. The left anterior descending artery was normal, and the left circumflex artery also had luminal irregularities. 

A review of her prior hospitalization records revealed that she underwent multiple coronary angiograms in the past and did not show any significant disease. Her serial troponin levels during the hospitalization were negative. Given the presence of angina-like chest pain, transient ischemic changes on EKG, and evidence of coronary vasospasm on coronary angiogram that resolved after administration of intracoronary nitroglycerin injection, we diagnosed the patient with Prinzmetal angina and opted for medical therapy with further optimization. She was discharged on isosorbide mononitrate 120 mg twice daily, ranolazine 1000 mg twice daily, and metoprolol tartrate 100 mg twice daily, and diltiazem 240 mg in lieu of her verapamil with a plan to follow up in the clinic.

## Discussion

Prinzmetal angina is caused by focal or diffuse coronary artery vasospasm, resulting in transient or persistent high-grade coronary artery obstruction. While the exact pathogenesis of coronary artery vasospasm has not been fully explained yet, multiple theories have been proposed in the medical literature. Risk factors and/or triggers such as smoking, alcohol use, stress, hyperventilation, and migraines in conjunction with mechanisms such as autonomic dysregulation via sympathomimetic and parasympathomimetic agents, vascular smooth muscle hypercontractility, endothelial dysfunction, magnesium deficiency, low-grade inflammation, oxidative stress, and gene polymorphisms have been reported to possibly contribute to coronary vasospasm in Prinzmetal angina patients [5]. Additionally, CAD also can predispose patients to Prinzmetal angina attacks; one study by Bertrand et al. estimated that 60% of their patients with obstructive coronary disease had superimposed Prinzmetal angina [6].

Since EKG changes are difficult to capture with the transient nature of Prinzmetal angina episodes, Sanjeeva et al. support the use of Holter monitoring to capture those instances, rule out certain arrhythmias that could be associated with syncope in Prinzmetal angina patients, and obviate the need for provocative testing [4]. However, cardiac catheterization with provocative testing is still important to consider as it can help to diagnose Prinzmetal angina and rule out underlying CAD as the cause [7]. 

Prinzmetal angina treatment is based on alleviating the acute attack, reducing the recurrence of additional attacks, and risk factor modification to reduce the capacity to trigger future episodes. Early treatment also helps prevent associated complications such as bradyarrhythmia, complete atrioventricular block, paroxysmal atrial fibrillation, ventricular tachycardia, ventricular fibrillation, and asystole [2]. As per the American Heart Association 2014 non-ST-segment elevation acute coronary syndrome guidelines, calcium channel blockers alone or in combination with long-acting nitrates have a class I recommendation as appropriate first-line agents for treating Prinzmetal angina [8]. 

The aforementioned therapies may be considered as initial options, but patients with refractory Prinzmetal angina, defined as persistent intermittent vasospasm despite being on a combination of two medications, need to be carefully evaluated as uncontrolled coronary vasospasm can put patients at risk for complications such as MI and sudden cardiac death. Nicorandil works via opening adenosine triphosphate-sensitive potassium channels, and it has been suggested as an option for refractory Prinzmetal angina [5, 9]. Based on the premise of microvascular spasm, possibly contributing to Prinzmetal angina, the use of ranolazine to successfully treat Prinzmetal angina has also been shown to be effective [10]. Stent implantation to treat Prinzmetal angina refractory to medical therapy has been found to be effective in some patients who are unresponsive to medical therapy alone [11]. Patients who undergo stent implantation should be carefully selected because there is some concern for in-stent restenosis, development of vasospasm in sections of the arteries adjacent to the stents, and concern for multivessel spasm. Surgical intervention via left stellate ganglion blockade has shown mixed results for refractory Prinzmetal angina [12]. 

Other treatment options have also been considered, but they may not have enough support to warrant inclusion in major guidelines, or the evidence regarding them is conflicting. Statins have been considered for preventing vasospasm. A study by Yasue et al. showed that statins combined with calcium channel blockers for six months reduced the number of patients with coronary spasm when they were assessed with repeat acetylcholine-based provocative testing [13]. However, conflicting evidence for statins exists; this requires further studies to validate their benefit in Prinzmetal angina. Alpha 1-adrenergic receptor antagonists such as prazosin have been considered as an alternative therapy, but non-selective beta-blockers should be avoided because they are a Prinzmetal angina risk factor and can allow for unopposed alpha-adrenergic vasoconstriction [5]. Aspirin, which may have value at low doses, has failed to show clear benefit with higher doses and even implicated, possibly contributing to coronary vasospasm [14]. Intracoronary administration of the Rho-kinase inhibitor fasudil helped to relieve coronary artery spasms induced by acetylcholine provocation, but further study is needed in Prinzmetal angina patients [15]. Bosentan and cilostazol have also been studied. Krishnan et al. showed that bosentan effectively treated Prinzmetal angina, but the high cost and drug-related adverse effects prevent high consideration of this medication [16]. Cilostazol was shown to be effective in treating Prinzmetal angina not well controlled with amlodipine [17]. However, further studies are necessary to validate its safety and efficacy as a long term medication. 

Prinzmetal angina has been considered separate from an acute coronary syndrome, but it is important to consider the overlap between these two processes as recurrent MI secondary to Prinzmetal angina has been reported and seen clinically on ventriculograms during coronary angiography [18, 19]. Iranirad and Sadeghi also describe a case with simultaneous multivessel coronary vasospasm that resulted in multisite MI and ventricular fibrillation [20]. 

Although the patient’s initial EKG did not show any ST-T wave changes, the EKG repeated after the patient complained of worsening chest pain showed the presence of new ST-T wave changes. Initially, suspicion was high for unstable angina due to a negative troponin level and chest pain characteristics. The development of ST elevations in the inferior leads on repeat EKG prompted cardiac catheterization, which ultimately helped differentiate atherosclerotic disease-related STEMI from Prinzmetal angina-related MI. Prinzmetal angina and MI could co-exist as in our case because the common underlying pathophysiological mechanism can be coronary artery spasm. In retrospect, this patient had a typical presentation of Prinzmetal angina with pain that started in the night, was non-exertional, and improved with nitroglycerin. Her prior history of Prinzmetal angina and smoking also were good clues. Considering the effect of underlying CAD on the development of Prinzmetal angina, prior catheterization information may be useful to predict the site of future attacks for which stenting might be considered if the patient does not have additional episodes in other sites or does not have a multivessel spasm. Additionally, the patient had multiple NSTEMIs in the past. However, the initial ECG and normal levels of cardiac enzymes masked her condition and could have caused warning fatigue similar to the story of the boy who cried wolf. Good medical reconciliation to assess medication compliance and accordingly adjust therapy may help decrease the future risk of Prinzmetal angina related MI.

## Conclusions

Prinzmetal angina presents with episodic chest pain and can present as a STEMI secondary to atherosclerotic plaque rupture on EKG. As Prinzmetal angina is often underdiagnosed, it is important to consolidate information from the timeline of chest pain, risk factors for coronary vasospasm, and initial evaluation upon first medical contact to correctly diagnose it. The risk of developing an MI from Prinzmetal angina should be greatly considered if the patient has a prior history of Prinzmetal angina and any of the aforementioned risk factors that predispose them to coronary vasospasm. The paucity of typical EKG changes on the initial evaluation of patients with suspected Prinzmetal angina is not sufficient to rule out this pathology as the underlying coronary vasospasm can be transient. In patients with high clinical suspicion that is contrary to information from EKGs, troponin levels, and other clinical elements, it may be imperative to use coronary angiography with an intracoronary agent such as nitroglycerin to differentiate between Prinzmetal angina and atherosclerotic plaque rupture related STEMI. In doing so, one can rule out alternative cardiac chest pain etiologies and avoid incorrect interventions such as stent placement if the vasospasm resolves with intracoronary vasodilator treatment and subsequent medical therapy.
